# Present in the Aquatic Environment, Unclear Evidence in Top Predators—The Unknown Effects of Anti-Seizure Medication on Eurasian Otters (*Lutra lutra*) from Northern Germany

**DOI:** 10.3390/toxics11040338

**Published:** 2023-03-31

**Authors:** Simon Rohner, Martina Gramer, Ivo Wiesweg, Oliver Scherf-Clavel, Peter Wohlsein, Martin Schmelz, Ursula Siebert, Franziska Richter, Manuela Gernert

**Affiliations:** 1 Institute for Terrestrial and Aquatic Wildlife Research, University of Veterinary Medicine Hannover Foundation, 30559 Hannover, Germany; 2 Department of Pharmacology, Toxicology and Pharmacy, University of Veterinary Medicine Hannover Foundation, 30559 Hannover, Germany; 3 Faculty of Chemistry, Aalen University, 73430 Aalen, Germany; 4 Department of Pathology, University of Veterinary Medicine Hannover Foundation, 30559 Hannover, Germany; 5 Aktion Fischotterschutz e.V, Otter-Zentrum Hankensbüttel, 29386 Hankensbüttel, Germany

**Keywords:** anti-seizure medication (ASM), Eurasian otter, top predator, pollution, biota monitoring, pharmaceuticals

## Abstract

Emerging contaminants are produced globally at high rates and often ultimately find their way into the aquatic environment. These include substances contained in anti-seizure medication (ASM), which are currently appearing in surface waters at increasing concentrations in Germany. Unintentional and sublethal, chronic exposure to pharmaceuticals such as ASMs has unknown consequences for aquatic wildlife. Adverse effects of ASMs on the brain development are documented in mammals. Top predators such as Eurasian otters (*Lutra lutra*) are susceptible to the bioaccumulation of environmental pollutants. Still little is known about the health status of the otter population in Germany, while the detection of various pollutants in otter tissue samples has highlighted their role as an indicator species. To investigate potential contamination with pharmaceuticals, Eurasian otter brain samples were screened for selected ASMs via high-performance liquid chromatography and mass spectrometry. Via histology, brain sections were analyzed for the presence of potential associated neuropathological changes. In addition to 20 wild otters that were found dead, a control group of 5 deceased otters in human care was studied. Even though none of the targeted ASMs were detected in the otters, unidentified substances in many otter brains were measured. No obvious pathology was observed histologically, although the sample quality limited the investigations.

## 1. Introduction

Rapid population growth, demographic changes towards aging populations, improvements in health care in many countries, and the continuous manufacturing of new drugs are reflected by increasing evidence of environmental contamination by pharmaceuticals and personal care products on a global scale [1,2]. Western European countries such as Germany in particular are anticipating a continuously growing demand for pharmaceutical consumption in the coming decades [3]. Insufficient disposal strategies enable pharmaceuticals and their metabolites to enter the aquatic environment via different routes, including urban sewage, hospital waste water, and animal husbandry [1,4,5]. Also referred to as micropollutants, they typically occur in the environment at low concentrations [6]. The most critical factor in regard to unintentional exposure is probably the persistent presence of all kinds of pharmaceuticals given their continuous use, leading to “pseudo-persistence” in the environment [7,8]. Such chronic exposure is believed to result in sublethal effects in aquatic wildlife, and is usually not taken into consideration during ecotoxicological assessments [9]. Together with parent compounds, different phases of metabolites end up in a cocktail of substances, the potential adverse effects of which on non-target species such as aquatic wildlife remain poorly studied [4,8,10,11,12]. It is difficult to predict the exposure to and uptake of pharmaceuticals in aquatic wildlife, due to the different chemical characteristics of specific substances [13]. Similar to humans, the ability of (wild) animals to metabolize certain drug doses varies according to age and sex, making predictions even more complex [8,14,15]. While the bioaccumulation of certain pharmaceuticals in aquatic wildlife has been demonstrated [10,13,16], the potential scope of biomagnification is not yet fully understood [13]. The known adverse impacts of pharmaceuticals on aquatic wildlife include indirect effects, such as reduced predator-avoidance behavior in fish due to antidepressants [17,18]. In addition, direct negative effects such as the feminization of fish due to synthetic estrogens [19,20], or impaired reproduction in amphibians due to progestagens [21], have been found. Although it is difficult to estimate consequences at the population level, a drastic example of the fatal non-target exposure of terrestrial wildlife to pharmaceuticals is evidenced by the massive decline of endangered vultures after scavenging on carrion contaminated with nonsteroidal anti-inflammatory drugs (NSAIDs) [22].

Designed to suppress epileptic seizures, anti-seizure medication (ASM) acts on different target molecules in the brain [23,24]. Grouped into first, second, and third generation of accreditation, ASMs can also have psychotropic effects on mood and behavior [24,25]. There are known side effects of ASMs in mammals, including reprotoxicity and teratogenicty in fetuses and newborns [26,27]. Pre- and postnatal exposure to certain ASMs can lead to fetal malformations, including in the central nervous system [28,29], or later cognitive dysfunction [24,30,31]. More specifically, phenobarbital, the oldest available ASM [24,26], was shown to negatively affect brain growth and behavior in mice (*Mus musculus* f. *domestica*) [32,33]. Certain doses of phenytoin lead to deficits in learning and hyperactivity in rats (*Rattus norvegicus* f. *domestica*) [34] as well as hyperexcitability in monkeys [26]. Furthermore, neurotoxicity at certain doses might occur [35]. Valproate (valproic acid) was shown to result in cerebellar hypoplasia [36] as well as functional impairment of behavior at subteratogenic concentrations in rodents [37]. Valproate is probably the ASM with the highest number of adverse effects on neurodevelopment in humans described to date [24,35]. Levetiracetam has been shown to be related to aggressive behavior in human patients with epilepsy [38]. Similar effects are suspected, but as yet not proven, for lamotrigine [39]. Overall, levetiracetam and lamotrigine are considered ASMs with relatively low risks of unwanted side effects in humans [24,35]. Neural tube defects in human newborns were thought to originate from prenatal exposure to carbamazepine [40]. More recent research has indicated that carbamazepine, among other ASMs, disrupts various neurotransmitters in teleost fish, resulting in different physiological changes [41]. Despite the difficulty of comparing the effects of drugs on humans and non-target species, there is evidence that a general comparison of brain development between species is possible [26,42]. Although the brain can be divided into different regions according to functional areas, all these parts are connected and function together via the components of a certain behavior that is displayed [43]. During neurogenesis, however, those regions develop in a temporally independent manner (including their own subpopulations of neurons) [26]. In the dentate gyrus of the hippocampal formation, neurogenesis continues until adulthood in humans, making it an important yet vulnerable region in the context of neuronal pathology [44,45]. Regulated by programmed cell death, only half of the initially created neurons will survive in an adult individual [46]. Acting via highly specific yet complicated and not fully understood mechanisms, interference in natural apoptotic processes might lead to increased cell death [26,47]. Studies on immature rodents showed that several ASMs may trigger apoptosis during neurogenesis, resulting in irreversible brain damage [48]. Moreover, the direct inhibition of the proliferation of neurons in the dentate gyrus of the hippocampal region due to phenobarbital was detected in innate rats, indicating the potential adverse impacts of ASMs on highly sensitive developmental processes of the growing mammalian brain [49].

At present, the Eurasian otter (*Lutra lutra*) is recovering from decades of population decline, and is currently recolonizing former habitats in many European countries, including Germany [50,51,52]. This is also the case in the northernmost federal state Schleswig-Holstein, where otters almost became extinct during the last century [50,53,54]. On a global scale, however, the Eurasian otter population is still showing a declining trend, and therefore remains strictly protected [55,56]. In Germany, otters are protected by national legislation [57] and are further listed in regional red list assessments [58,59]. Little is known about the overall health status of Eurasian otters in their range of distribution (see, for example, [60,61,62,63]), and only a few studies have covered aspects of population health in Germany so far [50,64,65,66]. Nevertheless, investigating the health of protected and elusive species such as otters is challenging, yet of utmost importance for successful conservation and species management, and post-mortem studies provide a valuable tool to assess various sources of information in this context [50,67,68]. Past and current research has revealed that otters, as top predators, are prone to the bioaccumulation of different pollutants, and therefore represent an excellent indicator species for the biota monitoring of the aquatic environment. For example, per- and polyfluoroalkyl substances (PFASs) have been detected in otters from the UK [69]; heavy metals have been investigated in otters from the UK and Denmark [70,71]; persistent organic pollutants (POPs) have been studied in otters from Sweden and the UK [72,73]; anticoagulant rodenticides have been found in otters from Germany (Regnery et al., in prep.); a wide-scope screening approach to search for various contaminants of emerging concern (CECs), including pharmaceuticals, has been applied to otters from different European countries [74]. Even though the simple presence of certain pollutants in otters does not necessarily prove adverse effects on their health, certain indications give reason for concern [75,76]. In particular, the related health effects of pharmaceutical contamination remain largely unknown in otters, and only three studies on this issue have been undertaken to date, to the best of the authors’ knowledge. In the first, the nonsteroidal anti-inflammatory drugs diclofenac and ibuprofen were detected in the fur of otters from the UK [77]. In another, blood and urine samples from Swedish otters were analyzed, and antidepressants (venlafaxine) and neuroleptics (risperidone), among other pharmaceuticals, were found [78]. In the last, analgesics (metamizole), among others, were detected in liver samples of otters from different European countries, including Germany [74]. While the presence of pharmaceuticals in otter tissue was proven in these studies, unfortunately, no risk assessments regarding the health of the study individuals could be undertaken.

In our present study, 20 wild Eurasian otters and 5 otters in human care were screened for potential contamination of the central nervous system by selected ASMs, using high-performance liquid chromatography (HPLC) and mass spectrometry (MS). While the wild otters were assumed to be potentially contaminated with ASMs via surface waters, the individuals under human care served as the control group. In addition to information derived from known medical records, including the euthanasia of captive animals, their aquatic environment (water chemistry) in the zoo was assumed to be more stable compared to wild individuals, with much larger home ranges and consequently a higher risk of contamination. The combination of screening for selected ASMs and post-mortem investigations, including histology, of otter brain samples represents our attempt to correlate the potential contamination of wildlife by pharmaceuticals and related pathology.

## 2. Materials and Methods

Brain samples from wild otters found dead in Schleswig-Holstein, Germany, were screened for selected ASMs via high-performance liquid chromatography (HPLC). Unknown peaks were additionally measured via mass spectrometry (MS). Deceased individuals under human care, known to have been euthanized using certain pharmaceuticals, served as a control group. Histological analyses were performed to correlate the potential contamination of wild otters with pathological findings in the central nervous system.

### 2.1. Carcass Collection

Wild otters from Schleswig-Holstein were collected between 2019 and 2020 under a population health research program, funded by the Ministry for Energy Transition, Climate Protection, Environment and Nature Agriculture, Environment, Nature, and Digitalization (MEKUN) [50]. Otters under human care originated from the Otter-Zentrum Hankensbüttel (OZ), Lower Saxony, Germany—a specialized zoo for mustelids run by the Aktion Fischotterschutz e.V. These animals lived in captivity and either died naturally or were euthanized due to serious illness. Except for three wild otters, whose brains were processed immediately after finding them, all remaining individuals from Schleswig-Holstein were stored frozen at −20 °C and thawed before necropsy. All otters under human care were stored frozen at −20 °C and thawed before necropsy.

### 2.2. Necropsy and Age Determination

All otters were necropsied following a special otter protocol, which has been extensively described previously [50]. Brain hemispheres were carefully extracted from the cranium using a hand saw, photographed, and weighed. Biopsy punches (Disposable Biopsy Punch, pfm medical ag, Cologne, Germany) were taken from both cerebral hemispheres and stored at −70 °C until further analysis. A lower or upper canine was taken during necropsy and processed for cementum aging [50,79]. For the captive individuals, birth dates were either known or assumed by the keepers.

### 2.3. Selected ASMs

In total, six ASMs were chosen for analysis in the otter brain samples: carbamazepine, lamotrigine, levetiracetam, phenobarbital, phenytoin, and valproic acid. While carbamazepine, phenytoin and phenobarbital are first-generation ASMs, lamotrigine, levetiracetam, and valproic acid are second-generation ASMs [23,24]. The methods of detection are well established at the Department of Pharmacology, Toxicology and Pharmacy (PTP), University of Veterinary Medicine Hannover, Foundation, Hannover, Germany [80].

### 2.4. Instrumental Analytics

HPLC was used to detect selected ASMs in wild otters and previously administered drugs in the captive otters’ brain samples. To try to identify unknown peaks measured via HPLC, MS was performed on some samples later on.

### 2.5. High-Performance Liquid Chromatography (HPLC)

Otter brain biopsies were mixed with H_2_O (1:3), homogenized (17.000/min) and aliquoted into subsamples of 200 µL. For precipitation, 200 µL of methanol or acetonitrile was added and samples were transferred onto a lab shaker for 5 min. Afterwards, samples were centrifuged for 20 min at 15,000 rpm and 4 °C. The supernatant was filtered with 0.2 µm centrifugal filters and again centrifuged for 20 min at 3500 rpm and 4 °C. Such prepared samples were stored at −30 °C until the final HPLC analysis.

Chromatic separation of the prepared otter brain samples was performed using an HPLC system (Shimadzu, Kyoto, Japan) with a Shimadzu LC-20 AT pump, a Shimadzu CBM-20A Prominence system controller, and a Shimadzu DGU-20A degasser unit. Sample components were detected using the Shimadzu SPD-20A UV detector. The stationary phase consisted of a first (EC 50/4 Nucleosil 120-5 C18) and second column (EC 250/4.6 Nucleosil 120-5 C18) (Macherey-Nagel, Düren, Germany). The mobile phase contained a mixture of phosphate buffer (pH 5.6) and acetonitril (68:32) to separate sample components at 35 °C. The wash solution consisted of acetonictril and H_2_O (80:20). Each sample was run for 13 min at a flow rate of 1.0 mL/min. Injection of 20 µL of sample solution into the HPLC was performed manually (detector wavelength 210 nm). A standard was established for carbamazepine, lamotrigine, levetiracetam, phenobarbital, phenytoin, and valproic acid before analyzing the brain samples (Figure S1). Limits of detection were derived from similar experiments with rats, as no blank samples for otters exist: carbamazepine 0.26 µg/g, lamotrigine 0.20 µg/g, levetiracetam 1.90 µg/g, phenobarbital 0.35 µg/g, phenytoin 1.05 µg/g, and valproic acid 5.00 µg/g. Further, a standard for pentobarbital was established, as at least one otter under human care was known to have been euthanized (Figure S1).

### 2.6. Mass Spectometry (MS)

For the non-targeted analysis, samples were analyzed using a liquid chromatography–high-resolution mass spectrometry (LC-HRMS) system consisting of a UHPLC Agilent Infinity II (Agilent Technologies Deutschland GmbH, Waldbronn, Germany) coupled to a qTOF X500R (AB Sciex LLP, Concord, ON, Canada). Chromatographic separation was performed using a Kinetex C18 column (2.6 µm particles, 50 × 2.1 mm) in gradient mode. Mobile phase A was composed of 10% (*v*/*v*) acetonitrile in water containing 5 mmol/L ammonium formiate and 0.1% (*v*/*v*) formic acid, whereas mobile phase B was made up of 90% (*v*/*v*) acetonitrile in water containing 5 mmol/L ammonium formiate and 0.1% (*v*/*v*) formic acid. The gradient started at 100% A and ended at 100% B after 30 min at a flow rate of 350 µL/min. Source conditions: Electrospray ionization in positive and negative modes using the TurbolonSpray-Interface at 5500 V, 300 °C, 25 psi curtain gas, 50 psi gas1 and gas2. MS parameters: CAD gas 7 AU, declustering potential 70 V with a spread of 10 V and a collision energy of 10 V. The system was operated in IDA (information-dependent acquisition) mode using TOF-MS and TOF-MS/MS as experiments. The mass range scanned was from 50 to 1000 *m*/*z*. The collision energy in the MS/MS experiments was 45 V with a spread of 15 V.

The homogenized samples were thawed on ice and extracted by protein precipitation. In brief, to 50 µL of homogenized sample, 200 µL of acetonitrile was added, which was shaken vigorously for 1 min. The sample was then centrifuged at 12 k rcf for 5 min at 4 °C. A total of 50 µL of the supernatant was diluted with 200 µL of water. Each sample was diluted in quintuplicate. In total, 10 µL of each dilution was injected into the system in a random order to minimize carry-over effects. The mass spectrometer was calibrated after every fifth run.

The data were analyzed using the Sciex OS software version 1.6 (Concord, ON, Canada) using the default settings for small molecules. The feature list (peaks detected) was sorted and inspected manually. The feature list was also searched for the most commonly observed pharmaceuticals in German surface waters based on compilations provided annually by the German Environment Agency (Maximum mean concentration >0.1 µg/L in the years 2015–2016, Article: https://www.umweltbundesamt.de/daten/chemikalien/arzneimittelrueckstaende-in-der-umweltzahl-der-wirkstoffe-in-human-und-tierarzneimitteln; assessed on 17 February 2022) and the database Pharmaceuticals in the Environment (https://www.umweltbundesamt.de/en/database-pharmaceuticals-in-the-environment-1, assessed on 17 February 2022).

### 2.7. Histology

The decomposition of the brains affected the successful extraction from the cranium during necropsy and sometimes resulted in damaged brain hemispheres. Therefore, only the macroscopically visible region of the hippocampal formation was targeted in the brains for thionine and immunofluorescence staining [81,82]. Standardized localizations of the cortex cerebri, the rostral brain stem including the basal ganglia, the cerebellum, the medulla oblongata and the regions of the thalamus, hypothalamus and hippocampus were covered for hematoxylin and eosin (HE) staining. The microscopical analysis focused on qualitative evaluation of potential neuronal degenerations, such as gliosis, cell dispersions, or cell loss, in all stainings.

### 2.8. Hematoxylin and Eosin

One brain hemisphere of each otter was stored in 10% buffered formalin during necropsy, as described by Rohner and colleagues [50]. Pituitary glands were sampled separately, directly transferred into embedding cassettes and stored in 10% buffered formalin together with the brain hemispheres. Formalin-fixed and paraffin-embedded slides were later prepared and stained with HE [83].

### 2.9. Thionine

Additionally, depending on the decomposition status, brain hemispheres were drained in 30% sucrose containing anti-fungal additive for 30 days to prevent freezing artefacts on the cryotome later. Brains were then rinsed with 0.1 M phosphate buffer and cut coronally into 0.5 cm sections using stirrup-shaped blades. The prepared brain sections were mounted on a freezing microtome (Reichert-Jung 1206) with connected cooling stage (Reichert-Jung Frigomobil) using a cryo compound. Slices of 40 µm were cut from each region, fixed on microscope slides coated with gelatin, and dried. Such prepared slides were Nissl-stained with thionine in the following order: descending dilution series of ethanol (95% > 75% > 50%), distilled water, thionine, ascending dilution series of ethanol (50% < 75% < 95%), isopropyl alcohol, and two times acetic acid n-butyl ester [84,85]. All slides were finally sealed with cover slips using a special mounting medium for histology. Analysis was performed using a microscope (Zeiss Axioscope 50) with a connected camera (Zeiss AxioCam color) and associated computer program (AxioVision, Special Edition 64, Rel. 4.9.1 SP1 (08-2013)).

### 2.10. Immunofluorescence

Slide preparation followed the same steps as described above for thionine. For staining, the sections were first rehydrated in 70% EtOH, then demasked by boiling the slides in citric acid buffer. After cooling them down in floating water, sections were blocked using a Bovine Serum Albumin (BSA) solution and then incubated with primary antibody solution overnight (dilution 1:500) (IBA1 Synaptic Systems 234004). Ionized calcium-binding adapter molecule 1 (IBA1) is a microglial and macrophage-specific calcium-binding protein. Resembling a commonly used protein marker of microglia activation, it is involved in the reorganization of the microglial cytoskeleton and in the support of the phagocytosis process. On the following day, sections were incubated in secondary antibody solution (dilution 1:500) (Alexa Fluor Af647 Invitrogen A21450). Antibodies were diluted in a self-prepared carrier solution (1:500) containing Tris-Buffered Saline (TBS), BSA, normal serum (Biozol LIN-ENG9010 (Histoserve) goat) and octylphenoxypolyethoxyethanol (triton ×100). After covering with ProLong™ Gold Antifade Mountant with DAPI (Cell Signaling Tech. 8961S), the sections were digitized with a Zeiss Axio Observer 7 microscope and associated computer program (Zeiss, Zen (blue edition (07-2017)) according to Käufer and colleagues [86].

### 2.11. Image Processing

The brightness and contrast of the images were partly adjusted in Adobe Photoshop 2022 version 24.1 (San José, CA, USA). In some cases, the background was manually removed with the same software.

## 3. Results

Data on locations, years of finding, sex, age, as well as pathological findings, excluding the brains, for all 20 wild otters from Schleswig-Holstein analyzed in this study have been extensively described elsewhere [50]. Four wild otters had been previously deskinned by taxidermists, and one otter in human care had an open abdomen and some internal organs were missing, which limited investigations.

### 3.1. Life History Data of Otters

Life history data of wild otters and otters under human care are given in Table 1.

Wild otters consisted of 10 male and 10 female individuals. One individual was younger than half a year, seven were between 0.5 and 2.5 years old, five were between 1.5 and 3.5 years old, two were between 2.4 and 4.5 years old, and the oldest individual was between 5.5 and 7.5 years old. Age was unknown for four wild otters. Animals originated from years 2016 (*n* = 1), 2017 (*n* = 1), 2018 (*n* = 2), 2019 (*n* = 8), and 2020 (*n* = 8). Nutritional status ranged between good (*n* = 13), moderate (*n* = 5), and poor (*n* = 2). Decomposition ranged between grade 1 (*n* = 1), grade 2 (*n* = 8), grade 3 (*n* = 9), and grade 4 (*n* = 2). Roadkill-induced trauma, resulting in lethal cardiac and circulatory failure, accounted for the death of the majority of wild otters (*n* = 17). Two otters died due to starvation and one individual due to bacterial septicemia after inhaling a foreign body (Table 1) [50].

Otters under human care consisted of two males and three females. Otters no. 22, 23 and 25 were taken into rehabilitation as wild orphaned cubs and originated from the Eastern German federal state of Brandenburg. Otters no. 21 and 24 were born in captivity (OZ). Except for one otter aged 2.3 years, all other individuals were older than 11 years, with the oldest one being 17.9 years old. They died in years 2003, 2012, 2014, 2019, and 2020, respectively. Nutritional status ranged from good (*n* = 2) to moderate (*n* = 2) and poor (*n* = 1). Decomposition grades ranged between 2 (*n* = 1), 3 (*n* = 3), and 4 (*n* = 1). No. 21 died due to metaplastic neoplasia, whereas no. 23 died in transit, probably due to cardiac arrest related to stress. No. 24 was found dead in its enclosure without any signs of disease. No. 22 died during anesthesia, whereas no. 25 was euthanized due to irreversible fusion of the spine (Table 1).

### 3.2. Instrumental Analytics

#### 3.2.1. High-Performance Liquid Chromatography

The brain samples were analyzed in batches: batch 1 (No. 1–9), batch 2 (No. 10–14, 21–25), and batch 3 (No. 15–20). None of the targeted ASMs—carbamazepine, lamotrigine, levetiracetam, phenobarbital, phenytoin, or valproic acid—were detected in any of the samples via HPLC. Instead, mostly unknown peaks were measured in 20 otters in total, including 15 wild and all 5 captive individuals. Peaks in otter no. 25 were identified as pentobarbital. The peaks in wild and captive individuals are depicted in Table 2 (for chromatograms of all otters, see Figures S2–S8).

#### 3.2.2. Mass Spectrometry

Otters from batches 1 and 2 (No. 1–9, 10–14 and 21–25) (Table 2) were chosen to be analyzed via MS, as some of those individuals showed unknown peaks in the HPLC (Table 3). Pentobarbital was identified in otter no. 25, which was known to be euthanized. Otters under human care therefore successfully served as the control group, and the administered drugs, taken from medical records, matched the HPLC and MS results. Additionally, two unknown substances were measured in some wild and captive otters (*n* = 13) via MS (Table 3). Those peaks did not match the peaks previously measured under HPLC analysis, and therefore no comparison between results from HPLC and MS was possible. Although no ultimate determination of the substances from MS could be achieved, possible sum formulas were calculated for substance 1 (C_2_H_27_N_19_O_3_; C_13_H_34_N_8_O_2_P; C_10_H_31_N_13_S; C_19_H_41_O_2_S_2_; C_18_H_37_O_7_; C_17_H_31_N_7_O_2_; C_19_H_33_N_4_O_3_; C_17_H_39_N_3_OS_2_; C_15_H_36_N_5_O_3_P; C_18_H_42_NP_3_; C_11_H_32_N_11_OP; C_12_H_33_N_10_OS; C_19_H_43_P_2_S; C_22_H_39_P_2_; C_16_H_35_N_3_O_6_; C_15_H_29_N_10_O; C_21_H_35_NO_4_; C_15_H_37_N_6_S_2_; C_17_H_38_N_2_O_4_P; C_18_H_40_NO_2_PS; C_9_H_30_N_14_P; C_14_H_35_N_7_O_2_S; C_22_H_37_O_2_S) and substance 2 (C_9_H_26_N_8_P; C_14_H_29_O_5_; C_13_H_23_N_7_; C_15_H_33_S_2_; C_15_H_25_N_4_O; C_11_H_28_N_5_OP). The calculated sum formulas were subject to a search in the PubChem database in order to check for any known drugs or pollutants. All possible structures were screened, but none of the results hinted towards a drug, drug metabolite, or pollutant. A comparison of fragment spectra with the spectra in the MassBank of North America (MoNA) database was also not successful. Thus, the compounds could not be assigned to drugs or known pollutants.

### 3.3. Histology

**Figure 1:**
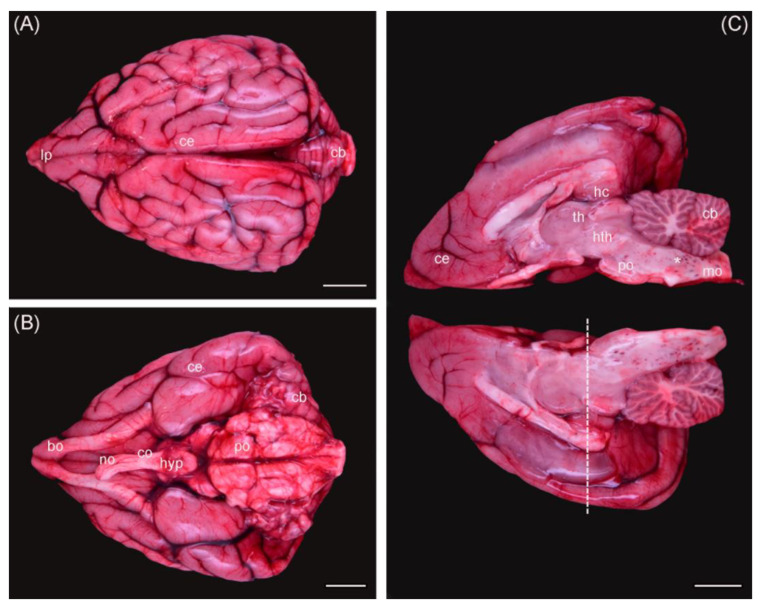
Otter brain dorsal view (**A**), ventral view (**B**), and medial view of both hemispheres (**C**). Ce = cerebrum; cb = cerebellum; bo = bulbus olfactorius; co = chiasma opticum; no = nervus opticus; hyp = hypophysis; po = pons; mo = medulla oblongata; th = thalamus; hth = hypothalamus; hc = hippocampus; * = hemorrhages. Dashed line = coronal section used for histology. Scale bar equals 1 cm in all views. The background was removed manually.

According to the grade of decomposition, 15 brains were considered for staining with HE, 11 brains for staining with thionine, and 1 brain exemplary for immunofluorescence. Macroscopically identified regions varied according to the shape of each individual brain hemisphere, which resulted in different locations and angles of section (Figure 1). This needed to be taken into consideration when comparing microscopical results later. Artefacts, caused by the process of freezing and thawing otters initially before necropsy, damaged brain cells and limited the possibility of detecting neurological alterations.

#### 3.3.1. Hematoxylin and Eosin (HE)

**Figure 2:**
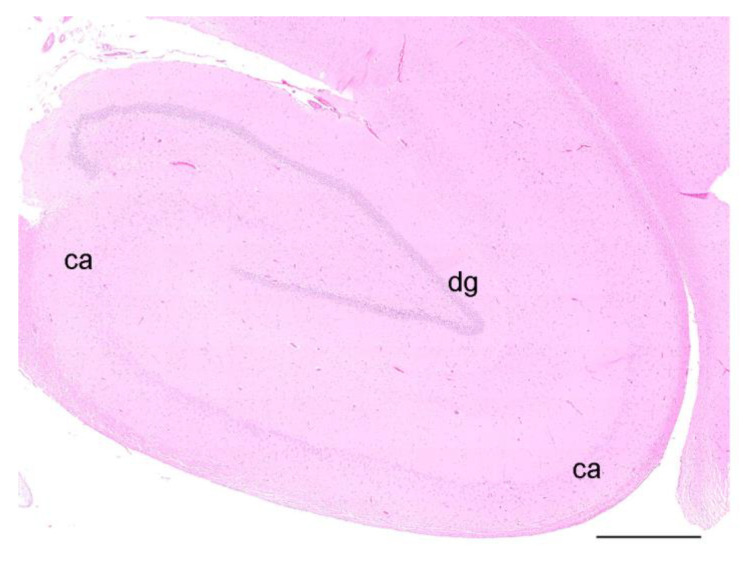
HE staining of the hippocampal formation of an otter, coronal section, showing the dentate gyrus (dg) and the cornu ammonis (ca). Scale bar equals 1 mm; the brightness and contrast have been modified.

Mild leptomeningeal and cerebellar hemorrhages occurred in two wild otters (No. 9, 13) and were attributed to blunt trauma, as both individuals were roadkills (not depicted). All other investigated otters did not show any morphological changes in the analyzed brain regions (No. 1, 2, 7–9, 10, 11, 14, 15, 17, 19–22) (Figure 2).

#### 3.3.2. Thionine

**Figure 3:**
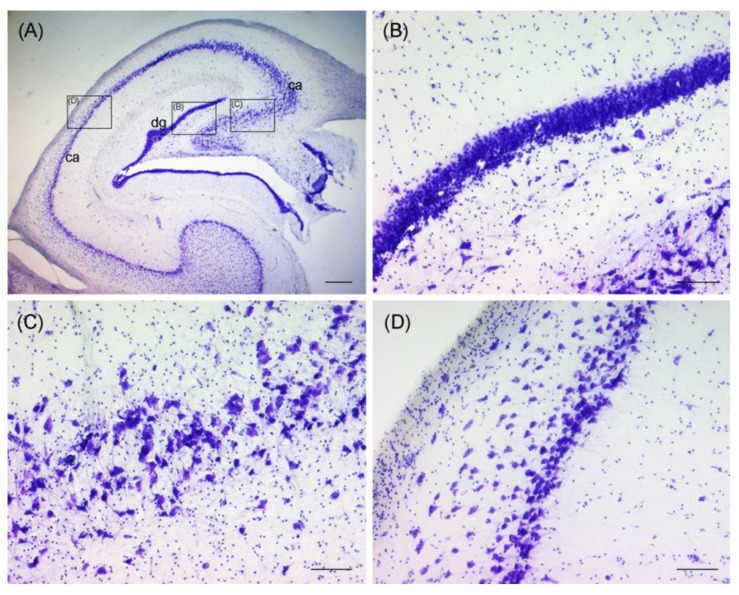
Thionine staining of the hippocampal formation of an otter, coronal section, showing the dentate gyrus (dg) and the cornu ammonis (ca) (**A**). Black rectangles indicate close-ups of (**B**–**D**). Granular cell layer of dentate gyrus is pictured in (**B**). Pyramidal cell layer of the cornu ammonis is pictured in (**C**,**D**). Scale bar equals 500 µm in (**A**) and 100 µm in (**B**–**D**). The brightness, contrast, and white balance have been modified in all images.

Ultimately, only the dorsal hippocampal formations of three wild otters, which were necropsied freshly and whose brains were therefore not frozen before fixation, could be analyzed microscopically (No. 13–15). Due to the lack of a specific brain atlas for otters and varying shapes of brain hemispheres, sections in the targeted regions differed and only limited comparison was possible (Figure 1). No morphological changes were observed in the analyzed regions of the brains of three animals (Figure 3).

#### 3.3.3. Immunofluorescence

**Figure 4:**
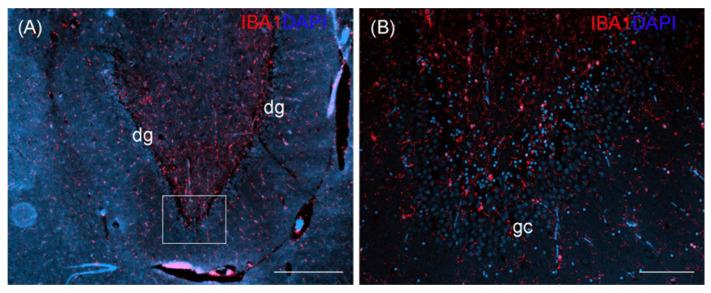
Ionized calcium-binding adapter molecule 1 (IBA1) staining of the hippocampal formation of an otter, coronal section, showing the dentate gyrus (dg) (**A**). White rectangle in (**A**) indicates the area of close-up (**B**) showing granular cells (gc). DAPI, 4′, 6-diamidino-2-phenylindole nuclear staining. Scale bar equals 500 µm in (**A**), 100 µm in (**B**).

An exemplary IBA1 staining is shown for wild otter no. 14 (Figure 4). Although we had no such staining from a non-wild control otter, the exemplary staining of the wild otter did not indicate gross morphological changes in the hippocampal formation.

## 4. Discussion

In this study, brains of 20 wild Eurasian otters and 5 individuals under human care were screened for potential contamination by ASMs and associated histopathological changes, indicating hypothesized adverse effects from pharmaceutical pollution on higher vertebrates in the aquatic environment. Neither were the targeted ASMs, nor was any overt neuronal pathology found in the investigated otter brains, whereas unidentified substances were measured. Different shortcomings limited investigations.

The previously selected ASMs carbamazepine, lamotrigine, levetiracetam, phenobarbital, phenytoin and valproic acid were chosen as target substances to reflect the expected increase in use of this substance class in Germany in the future [87]. While limited knowledge is available about the persistence and degradation of ASMs in the aquatic environment [88,89], carbamazepine has been comparatively well studied and proven rather stable in past experiments [90,91]. It was even proposed as an anthropogenic marker for water quality, given its widespread occurrence in water testing schemes [92,93]. Previous chemical monitoring of surface waters in Germany further confirmed that ASMs rank among the pharmaceutical classes detected most often in the aquatic environment [3,4,94,95]. In the northernmost German federal state of Schleswig-Holstein, however, only carbamazepine is assessed during the routine monitoring of streams [96]. In 2021, the ASMs carbamazepine and lamotrigine were additionally measured downstream of sewage water treatment plants, resulting in widespread concentrations above the limit of quantification (A. Kock, personal communication on 29 March 2022). The general occurrence of ASMs in streams and consequently the potential exposure of otters was therefore assumed.

A total of 20 deceased wild otters that were investigated within a population health-monitoring program in Schleswig-Holstein and originated from years 2019–2020 served as the study material in terms of brain samples [50]. HPLC failed to detect any of the selected ASMs in those wild otter brains, whereas random peaks indicated the presence of unknown substances in many of the samples. In addition to the wild specimens, five otters from a zoo were analyzed for the same ASMs. None of the targeted ASMs was detected in any of those individuals either. Those otters under human care were chosen as a control group, as their environment in the enclosures was assumed to be more stable in terms of water chemistry compared to the unpredictable conditions for wild otters, which tend to have large home ranges [97], and thus seemed more susceptible to potential contamination with ASMs. Furthermore, one otter under human care was known to have been euthanized using pentobarbital. A barbiturate similar to the ASM phenobarbital, pentobarbital produced a clear peak in the HPLC at minute 11.125 in the standard chromatogram. As the peaks of the euthanized otter originated at minutes 10.662 and 12.131, respectively, varying retention times needed to be taken into consideration due to the high concentration of pentobarbital in the sample. Additionally, the standard chromatograms using methanol slightly differed from those using otter brain tissue as a matrix for ASMs. The peaks in the euthanized otter were therefore identified as pentobarbital, confirming that the method itself was correct and could be used to detect the targeted substances. According to the analyses undertaken in three different batches, otters no. 1–9 represented one batch and all showed a random peak at a minute 6. Contamination of this batch needed to be taken into consideration when looking at such a distinct pattern. Further random peaks were measured in both of the other batches, albeit less frequently and more randomly distributed. Several individuals produced peaks at minutes 2–5. Contamination or brain-specific compounds most likely explain those early peaks, whereas it was also possible that these peaks indicate other unknown substances. Otters no. 22 and 25 had been reportedly anesthetized previously, whereas no. 22 died during this process and no. 25 was euthanized under anesthesia. Whether peaks at minutes 10 and 12 represent anesthetic compounds is not clear, as specific information on substances could not be retrieved from the medical records of those animals. Likewise, contamination could not be excluded for the other random peaks in the latter two batches.

In addition to HPLC, MS was conducted for batches 1 and 2 in an attempt to identify some of the previously measured random peaks. Alongside the selected ASMs, other known pharmaceuticals measured in German chemical monitoring programs [94], and information on other pharmaceuticals measured in the chemical monitoring of the federal state of Schleswig-Holstein, as described previously, were taken into consideration for the measured peaks. Except for pentobarbital in otter no. 25, none of the targeted substances could be found in any of the screened samples via MS, and limitations in detecting certain compounds or metabolites, other than the ASMs under focus, need to be taken into consideration. On the contrary, two different peaks at minute 2 indicated the potential presence of two unidentified substances in the samples. When compared to the HPLC results, the peaks from both methods did not seem to match. As discussed for HPLC, contamination of the samples did not seem unlikely. Nevertheless, the fact that MS failed to detect the selected pharmaceuticals or their metabolites did not prove their absence in the samples.

When performing post-mortem investigations of wildlife such as otters, histology offers excellent abilities for examining morphological alterations of organs on a microscopical level [50]. Histological analysis provides a tool to detect lasting tissue alterations, even after elimination of contaminants from the brain. In this first attempt, the authors wanted to determine whether brain histology would prove to be useful in assessing ASM-induced adverse effects on higher aquatic vertebrates, like otters. Certain ASMs, as single substances or as a combination of substances, are known to cause neuronal lesions in mammals during development, such as microscopically visible neuronal cell loss, associated with the loss of neurotrophins and certain proteins [47]. The hippocampal formation in particular, including the region of the dentate gyrus, was found to react with neuronal apoptosis during development, resulting in reduced cell densities [48]. However, ASMs in the assumed concentrations present in water were not expected to result in overt morphological alterations in the brain tissue of mostly subadult or adult otters. Activated microglia cells are a very sensitive measure used to detect even subtle impacts on brain tissue, but this requires tissue of at least moderate quality. Nevertheless, it was also deemed important to attempt assessments of brain histology to characterize the tissue and to exclude obvious pathology such as tumors or encephalitis, which could have influenced the blood–brain barrier and the accumulation of pharmaceuticals in the otters. Furthermore, our goal was to demonstrate whether brain samples from otters displaying advanced decomposition might still be useful for histological analysis, thereby motivating the careful collection and storage of all carcasses found for future research on pharmaceutical residue analysis. While most of the brains (*n* = 15) were used for HE staining, interpretation was limited by the quality of the slides due to freezing artefacts and the decomposition status of the original otter carcasses. Likewise, only 3 of 11 thionine-stained brains could be analyzed histologically. Notably, those three brains originated from the three otters that were sampled fresh, shortly after being found, and their brains were not frozen. Freezing therefore turned out to be the most limiting factor for thionine and immunofluorescence assays in this study. In addition to HE and thionine, IBA1 was used to make a rough estimation of putative microglia activation. None of the stains revealed any evidence of neuronal pathology, such as cell dispersions, cell loss or gliosis, in the studied otters, when using a ferret (*Mustela putorius*) brain atlas [81] for comparison, as the ferret is a close relative in the family of the mustelids [98].

Most otters used for post-mortem research in northern Germany are roadkills, usually displaying severe traumatic lesions, often including fractured skulls and damaged brains. The rather low sample size in this study originated from two years of otter monitoring in Schleswig-Holstein and represents the natural limitations of working with a strictly protected species [50]. It cannot be excluded that the rather low and biased sample size further limited the chances of detecting ASMs. Additionally, the age of the investigated otters was not homogeneously distributed, as most individuals represented subadults, and only one cub and one otter older than five years old were integrated. Especially when taking into account the reprotoxicity and teratogenicity of certain ASMs during development [26,27,28], the investigation of more otter cubs would have been useful. Unfortunately, the brain hemispheres of the only otter cub in this study dissolved during fixation, and could not be used for histological analysis. On the other hand, Jacobs and colleagues [44] and Kempermann [45] described the continuation of neurogenesis into adulthood in certain regions of the central nervous system, raising the question of whether it would be more efficient to investigate adult otters when it comes to pharmaceutical pollution and expected neuropathology. The brain of the oldest wild individual in our study, a male otter aged 5.5–7.5 years, unfortunately was already too decomposed to be used for histological analysis.

Wild otters were compared with five individuals under human care in this study. Importantly, three of the five otters under human care were found abandoned in the wild and were taken into rehabilitation as cubs. Therefore, pre- and postnatal exposure to pharmaceuticals could not be excluded for those individuals. Even though their enclosures were partly filled with water from surrounding streams, the water chemistry was assumed to be more stable compared to the large home range, and thus broad exposure to water bodies by wild otters [97]. Still, it could not be excluded that even the two captive-born otters had experienced some sort of pharmaceutical pollution throughout their lifetime, as no monitoring of pharmaceuticals in the inflowing streams to the zoo was routinely conducted. As ASMs are present in surface waters in Germany [3,4,94,95] and consequently the chronic exposure of otters can be assumed, it remained unclear why none of the targeted ASMs could be traced in the otters. Bean and colleagues [99] hypothesized that otters are especially susceptible to the bioaccumulation of pharmaceuticals in their aquatic environment, and other studies have previously proven their exposure to certain drugs [74,77,78]. Richards and colleagues [77] were able to detect certain pharmaceuticals in the fur of Eurasian otters from the UK. Otter fur is extremely dense and provides the primary insulation barrier for the species [100]. Grooming their fur is a behavior readily observed in Eurasian otters [97]. Even though one could assume the fur might function as a barrier for the otters and reduce dermal resorption from micropollutants, grooming and following oral uptake need to be taken into consideration as a potential port of entry for environmental pollutants [101]. Studies on the pharmacokinetics of AMSs, for example of carbamazepine, showed dose- and species-specific differences regarding oral bioavailability in animal models using rats, dogs, and humans [102]. Whereas oral bioavailability was determined at 40% in rats [103] and 91% in dogs [104], it was defined at 75–85% in humans [105]. Still, experimental settings, such as fasted animals and dosing, need to be taken into consideration [102,103,104,105]. ASMs are typically small lipophilic molecules designed to achieve good brain penetration passively, but some depend on active transport to pass the blood–brain barrier [24]. For example, the brain/plasma ratio was determined at 1.5 for carbamazepine in humans [106], representing good brain penetration and accumulation. For valproate, in comparison, the brain/plasma ratio was assessed at only 0.20–0.25 in rats ([107]; Gernert personal communication on 27 February 2023), which is related to its high plasma protein-binding ability. Plasma protein binding differs between ASMs and target organisms [103,108]. In addition, studies on the plasma half-lives of ASMs showed marked interspecies differences, by typically showing much shorter half-lives in dogs compared to humans, for example [109]. The surface water concentrations of ASMs in German waters may be too low to result in measurable concentrations in the brains of otters. Still, no data on the potential neurotoxicity of ASMs in otters exist to date. Given that marked interspecific pharmacokinetic differences occur between, for example, humans and dogs [109], comparing the effects of acute, therapeutic dosages in dogs with assumed chronic, subtherapeutic dosages in wild otters remains difficult. A further limitation is thus the fact that metabolites of the selected ASMs were not included in the analyses of this study. In addition, abiotic factors, including possible in-stream attenuation, differ among micropollutants and will affect their concentrations in surface waters [92]. No data exist regarding the stability of ASMs in otter carcasses that might remain at their initial place of death in the environment for hours or days, until the carcass is recovered for necropsy.

A challenge when assessing potential effects of micropollutants such as ASMs on aquatic organisms is the heterogeneity of past experimental settings (short-term vs. long-term), the different study organisms (ranging from invertebrates to fish), and the varying endpoints (behavior, physiology, etc.) [110,111]. The fact that no morphological alterations were seen in the studied otter brains could also point towards more subtle, difficult to detect pharmacological effects, such as on neurotransmission in otters, as seen for example in teleost fish [41]. Taking into account that even subtle side effects of certain drugs may be of low relevance in humans, they may, in contrast, be fatal in wild animals that rely on their full physiological and behavioral potential for survival [8,19,41]. Whether or not such subclinical effects might have significant health impacts on aquatic wildlife remains difficult to say, as limited evidence complicates interpretation either way [110]. In addition to brain samples, other organ tissue might have been useful for the comparison of ASM accumulation in the studied otters. Future studies on the pharmaceutical contamination of top predators such as Eurasian otters could benefit from wide-scope target-screening programs, wherein a great variety of environmental pollutants can be assessed simultaneously, giving a better idea of which compounds, including their metabolites, are to be expected in certain organs in targeted wildlife species [74].

## 5. Conclusions

More research is needed to shed further light on the little-known effects of unintentional medication of the aquatic environment. Both direct and indirect effects on potentially contaminated wildlife need to be taken into consideration. Post-mortem investigations of top predators offer valuable insights into population and ecosystem health. Depending on the study design, the storage and decomposition of investigated wildlife may be a limiting factor. Due to their position in the trophic chain, these predators are prone to the bioaccumulation of pollutants, and are therefore highly useful for biota monitoring programs. Future post-mortem studies on pharmaceuticals and related health effects in higher vertebrates such as otters could benefit from wide-scope target-screening programs in order to preselect pharmaceuticals and metabolites with good chances of detection.

## Figures and Tables

**Table 1:** Life history data and cause of death of all wild otters (w) from Schleswig-Holstein, Germany and five otters under human care (hc) used in the study.

Otter No.	Year of Finding/ Death	Sex	Age (Years)	Status before Necropsy	Decompo-sition Grade	Nutritional State	Cause of Death
1 (w)	2016	m	2.5–3.5	frozen	2	moderate	trauma
2 (w)	2017	m	0.75–2.5	frozen	3	good	trauma
3 (w)	2018	f	-	frozen	2	moderate	trauma
4 (w)	2018	f	1.3–2.25	frozen	3	moderate	trauma
5 (w)	2019	f	-	frozen	3	good	trauma
6 (w)	2019	f	0.6–1.7	frozen	3	good	trauma
7 (w)	2019	m	-	frozen	3	moderate	bacterial septicemia
8 (w)	2019	f	1.5–2.5	frozen	2	moderate	trauma
9 (w)	2019	f	2.5–3.5	frozen	4	good	trauma
10 (w)	2019	m	0.5–2.5	frozen	4	good	trauma
11 (w)	2019	f	2.4–4.5	frozen	2	good	trauma
12 (w)	2019	f	2.3–3.3	frozen	3	good	trauma
13 (w)	2020	m	-	fresh/frozen	2	good	trauma
14 (w)	2020	m	1–2	fresh/cooled	3	good	trauma
15 (w)	2020	m	3.25–4.25	fresh/cooled	1	bad	starving
16 (w)	2020	m	5,5–7,5	frozen	2	good	trauma
17 (w)	2020	f	2.3–3.3	frozen	2	good	trauma
18 (w)	2020	m	0	frozen	2	bad	starving
19 (w)	2020	f	0.5–2.5	frozen	3	good	trauma
20 (w)	2020	m	1.5–3.5	frozen	3	good	trauma
21 (hc)	2003	m	11.2	frozen	4	good	metastatic neoplasia
22 (hc)	2012	f	2.3	frozen	3	good	anesthesia
23 (hc)	2014	f	11.25	frozen	3	moderate	cardiac arrest
24 (hc)	2019	f	17.9	frozen	3	bad	unknown
25 (hc)	2020	m	6.75–7	frozen	2	moderate	euthanasia

**Table 2:** Substances in the HPLC in 15 wild otters (w) and 5 otters under human care (hc). Absence of any substance is indicated (-).

No. Otter	Peak Substance 1 (min)	Peak Substance 2 (min)	Peak Substance 3 (min)	Peak Substance 4 (min)	Peak Substance 5 (min)	Peak Substance 6 (min)	Peak Substance 7 (min)	Peak Substance 8 (min)
1 (w)	2.013	-	-	6.909	-	-	-	-
2 (w)	2.020	-	-	6.913	-	-	-	-
3 (w)	-	-	-	6.909	-	-	-	-
4 (w)	-	-	-	6.653; 6.906	-	-	-	-
5 (w)	-	-	-	6.653; 6.906	-	-	-	-
6 (w)	-	-	-	6.913	-	-	-	-
7 (w)	2.144	-	-	6.905	-	-	-	17.002
8 (w)	2.037	-	-	6.670; 6.922	-	-	-	-
9 (w)	2.029	-	-	6.919	-	-	-	-
10 (w)	-	-	-	-	9.700	-	-	-
11 (w)	-	4.489	-	-	-	-	-	-
12 (w)	-	4.459	-	-	-	-	-	-
13 (w)	-	4.499	5.423	-	-	-	-	-
14 (w)	-	4.482	-	-	-	-	-	-
15 (w)	-	4.523	-	-	-	-	-	-
21 (hc)	-	-	-	-	-	10.670	-	-
22 (hc)	-	-	-	-	-	-	12.097	-
23 (hc)	-	4.498	-	-	-	-	-	-
24 (hc)	-	4.474	-	-	-	-	-	-
25 (hc)	-	-	-	-	-	10.662	12.131	-

**Table 3:** Substances 1 and 2 in the mass spectrometry in wild otters (w) and otters under human care (hc). Presence (+) and absence (-) of any substance is indicated.

No. Otter	Substance 1, Retention Time 2.19 min; *m*/*z* Accurate *m*/*z* 365.2531	Substance 2, Retention Time 2.15 min; Accurate *m*/*z* 277.2006
4 (w)	-	+
5 (w)	+	-
7 (w)	+	-
8 (w)	+	+
11 (w)	+	+
12 (w)	+	+
13 (w)	+	+
14 (w)	+	+
21 (hc)	+	+
22 (hc)	+	+
23 (hc)	-	+
24 (hc)	+	+
25 (hc)	+	+

## Data Availability

Data is contained within the article or Supplementary Material.

## Supplementary Materials

The following supporting information can be downloaded at: https://www.mdpi.com/article/10.3390/toxics11040338/s1, Figure S1: Standard of ASMs lamotrigine (peak at min. 5.301), phenobarbital (peak at min. 6.470), valproic acid (peak at min. 9.752), carbamazepine (peak at min. 10.287), and phenytoin (peak at min. 10.551) (A). Standard chromatogram of ASM levetiracetam (peak at min. 7.308) (B). Standard chromatogram of pentobarbital (peak at min. 11.125) (C). Figure S2: Chromatogram of otter no. 1 with peaks at min. 2.013 and 6.909 (A), of otter no. 2 with peaks at min. 2.020 and 6.913 (B), of otter no. 3 with a peak at min. 6.909 (C), and of otter no. 4 with peaks at min. 6.653 and 6.906 (D). Figure S3: Chromatograms of otter no. 5 with peaks at min. 6.653 and 6.906 (A), of otter no. 6 with a peak at min. 6.913 (B), of otter no. 7 with peaks at min. 2.144, 6.905 and 17.002 (C), and of otter no. 8 with peaks at min. 2.037, 6.670 and 6.922 (D). Figure S4: Chromatograms of otter no. 9 with peaks at min. 2.029 and 6.919 (A), of otter no. 10 with a peak at min. 9.700 (B), of otter no. 11 with a peak at min. 4.489 (C), and of otter no. 12 with a peak at min. 4.459 (D). Figure S5: Chromatograms of otter no. 13 with peaks at min. 4.499 and 5.423 (A), of otter no. 14 with a peak at min. 4.482 (B), of otter no. 15 with a peak at min. 4.523 (C), and of otter no. 16 (D). Figure S6: Chromatograms of otter no. 17 (A), of otter no. 18 (B), of otter no. 19 (C), and of otter no. 20 (D). Figure S7: Chromatograms of otter no. 21 with a peak at min. 10.670 (A), of otter no. 22 with a peak at min. 12.097 (B), of otter no. 23 with a peak at min. 4.498 (C), and of otter no. 24 with a peak at min. 4.474 (D). Figure S8: Chromatogram of otter no. 25 with peaks at min. 10.662 and 12.131.
