# Supplementary material for: Present in the Aquatic Environment, Unclear Evidence in Top Predators—The Unknown Effects of Anti-Seizure Medication on Eurasian Otters (Lutra lutra) from Northern Germany
Source: Toxics. 2023 Mar 31;11(4):338. doi: 10.3390/toxics11040338 (PMC10142713; doi:10.3390/toxics11040338)
Supplement: Supplementary file 1 [file toxics-11-00338-s001.zip › toxics-2290315-supplementary.pdf]

Supplementary materials

Standard chromatograms of anti-seizure medications (ASMs) targeted in the study and standard chromatogram of pentobarbital

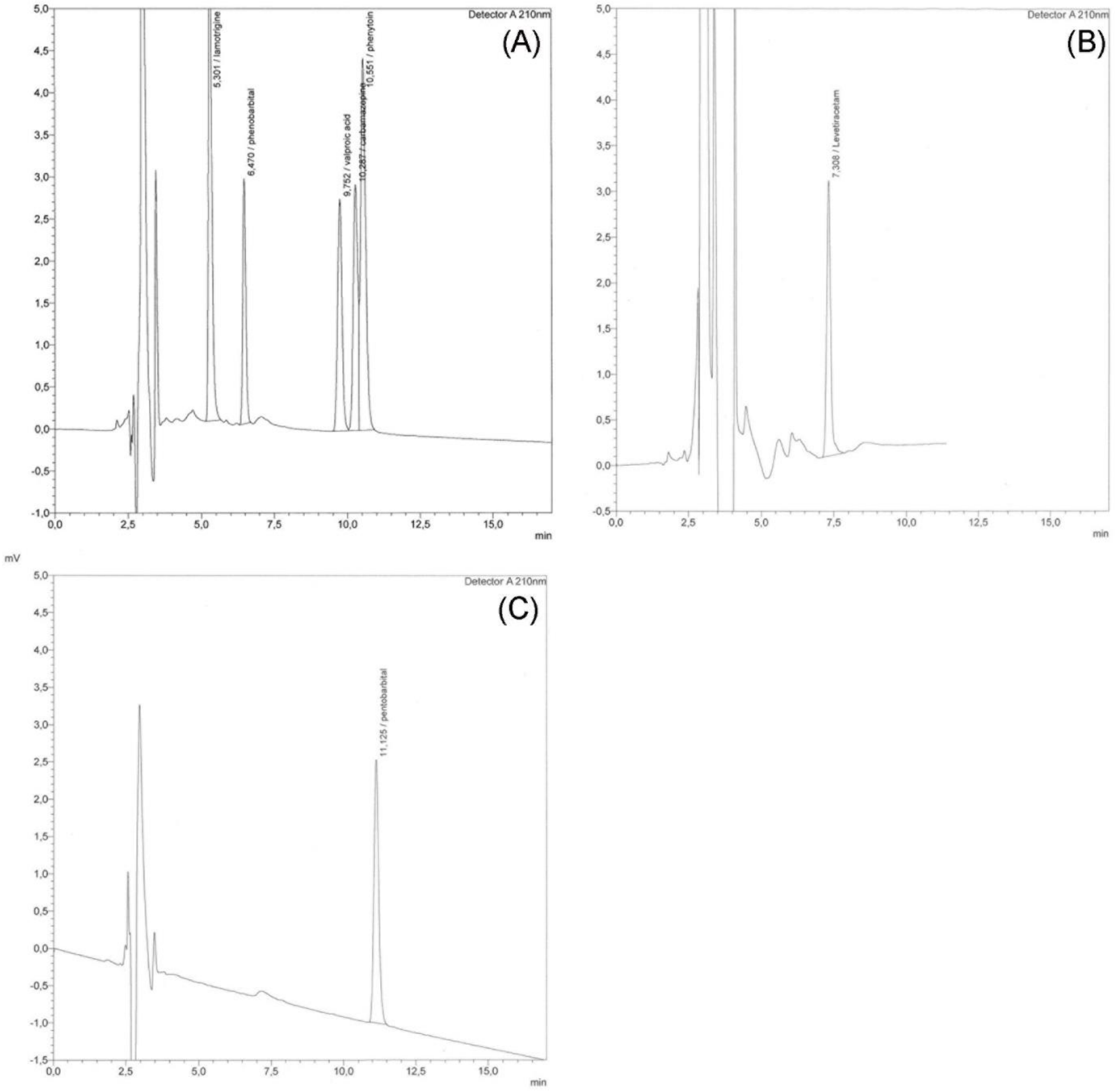

Figure S1: Standard of ASMs lamotrigine (peak at min. 5.301), phenobarbital (peak at min. 6.470), valproic acid (peak at min. 9.752), carbamazepine (peak at min. 10.287), and phenytoin (peak at min. 10.551) (A). Standard chromatogram of ASM levetiracetam (peak at min. 7.308) (B). Standard chromatogram of pentobarbital (peak at min. 11.125) (C).

Chromatograms of wild otters no. 1 – 20 used in the study

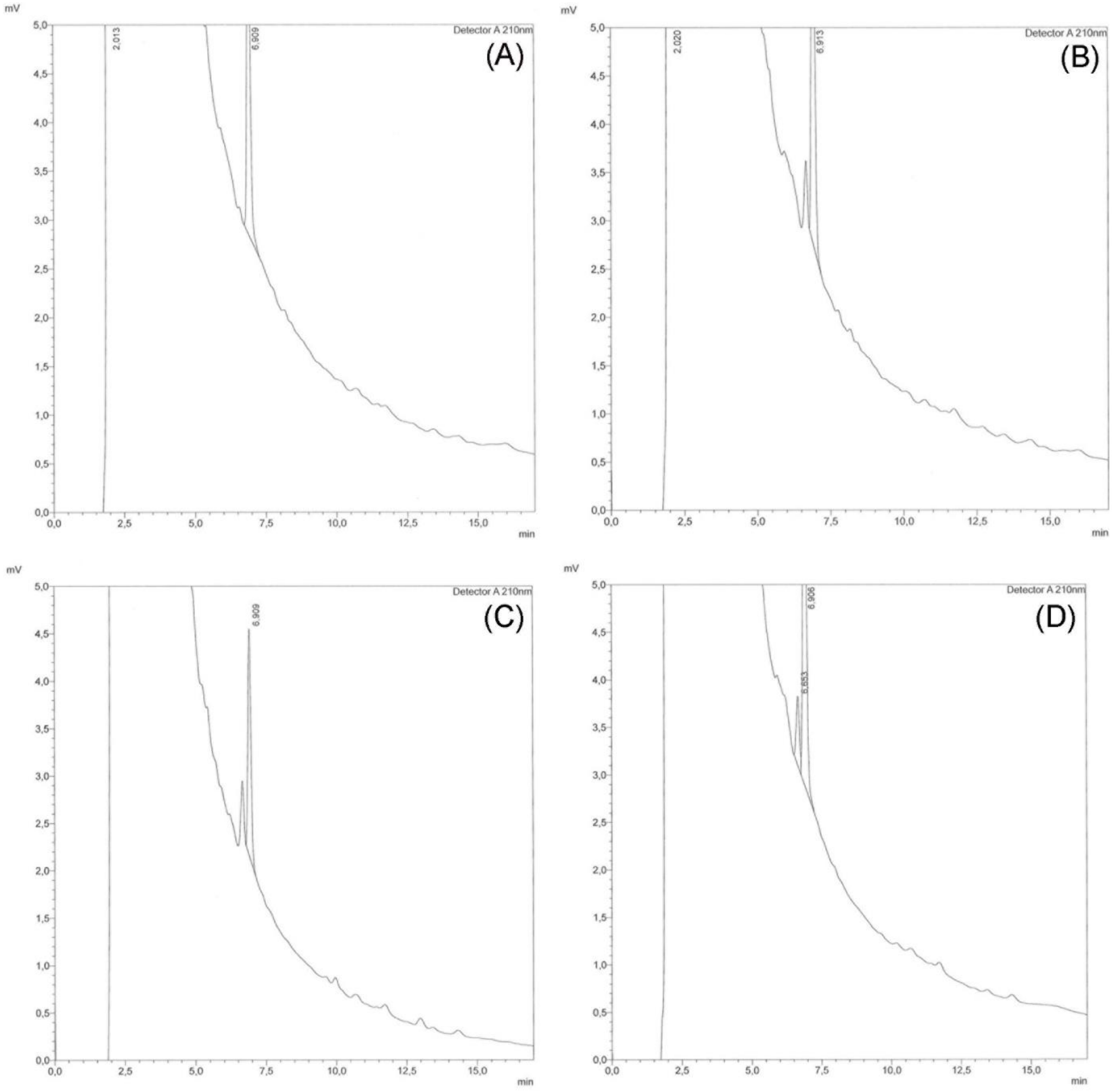

Figure S2: Chromatogram of otter no. 1 with peaks at min. 2.013 and 6.909 (A), of otter no. 2 with peaks at min. 2.020 and 6.913 (B), of otter no. 3 with a peak at min. 6.909 (C), and of otter no. 4 with peaks at min. 6.653 and 6.906 (D).

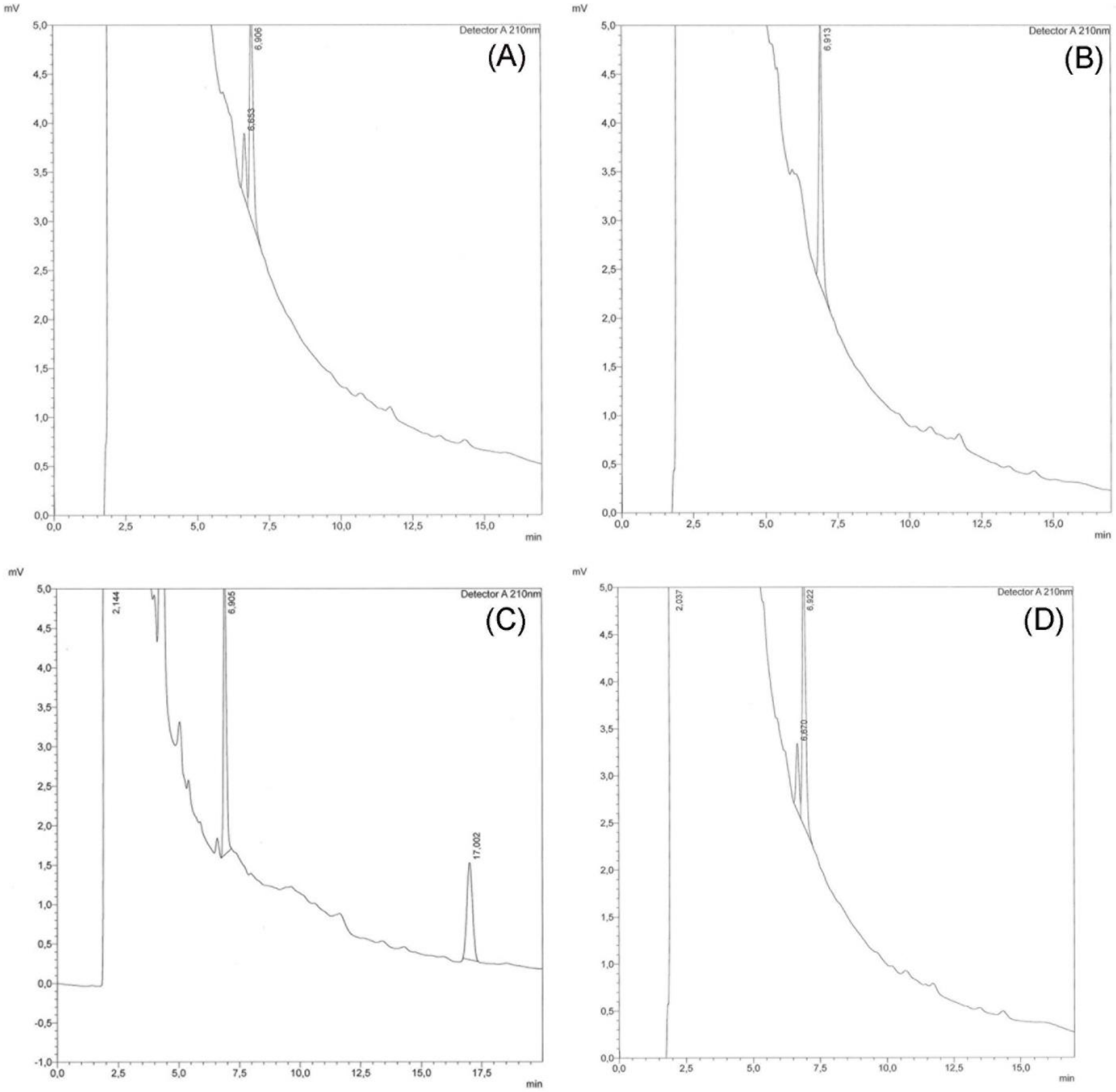

Figure S3: Chromatograms of otter no. 5 with peaks at min. 6.653 and 6.906 (A), of otter no. 6 with a peak at min. 6.913 (B), of otter no. 7 with peaks at min. 2.144, 6.905 and 17.002 (C), and of otter no. 8 with peaks at min. 2.037, 6.670 and 6.922 (D).

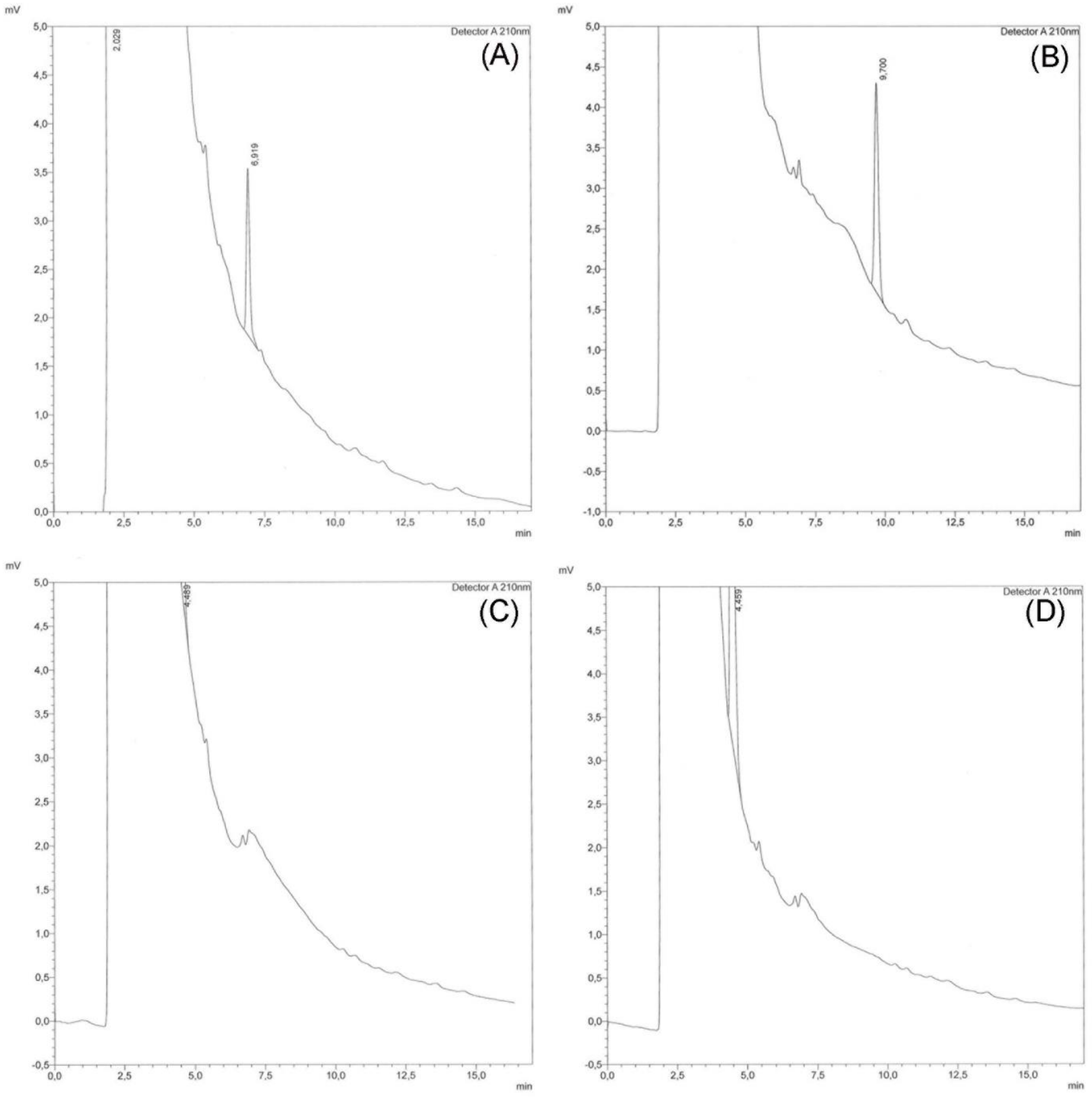

Figure S4: Chromatograms of otter no. 9 with peaks at min. 2.029 and 6.919 (A), of otter no. 10 with a peak at min. 9.700 (B), of otter no. 11 with a peak at min. 4.489 (C), and of otter no. 12 with a peak at min. 4.459 (D).

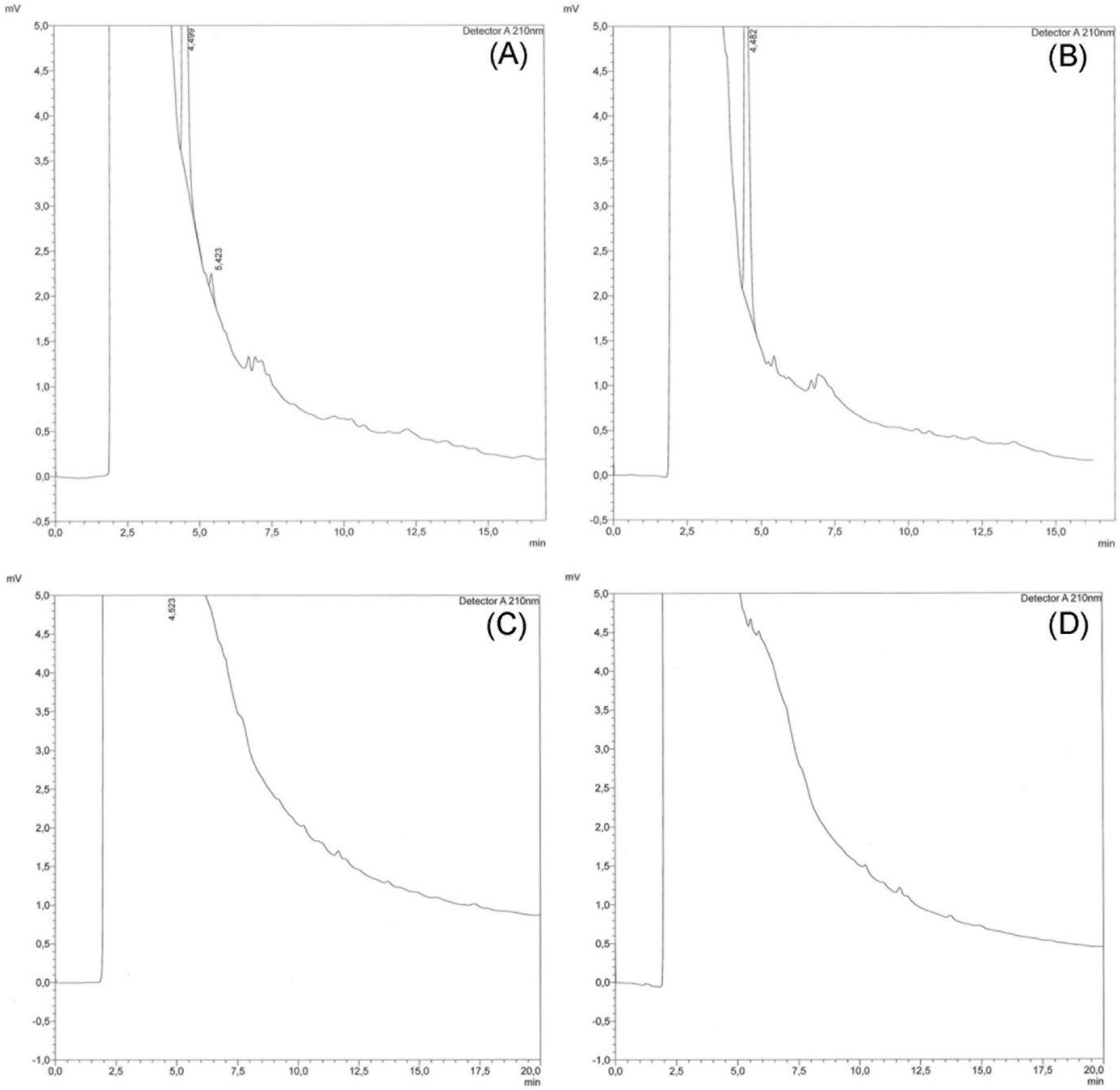

Figure S5: Chromatograms of otter no. 13 with peaks at min. 4.499 and 5.423 (A), of otter no. 14 with a peak at min. 4.482 (B), of otter no. 15 with a peak at min. 4.523 (C), and of otter no. 16 (D).

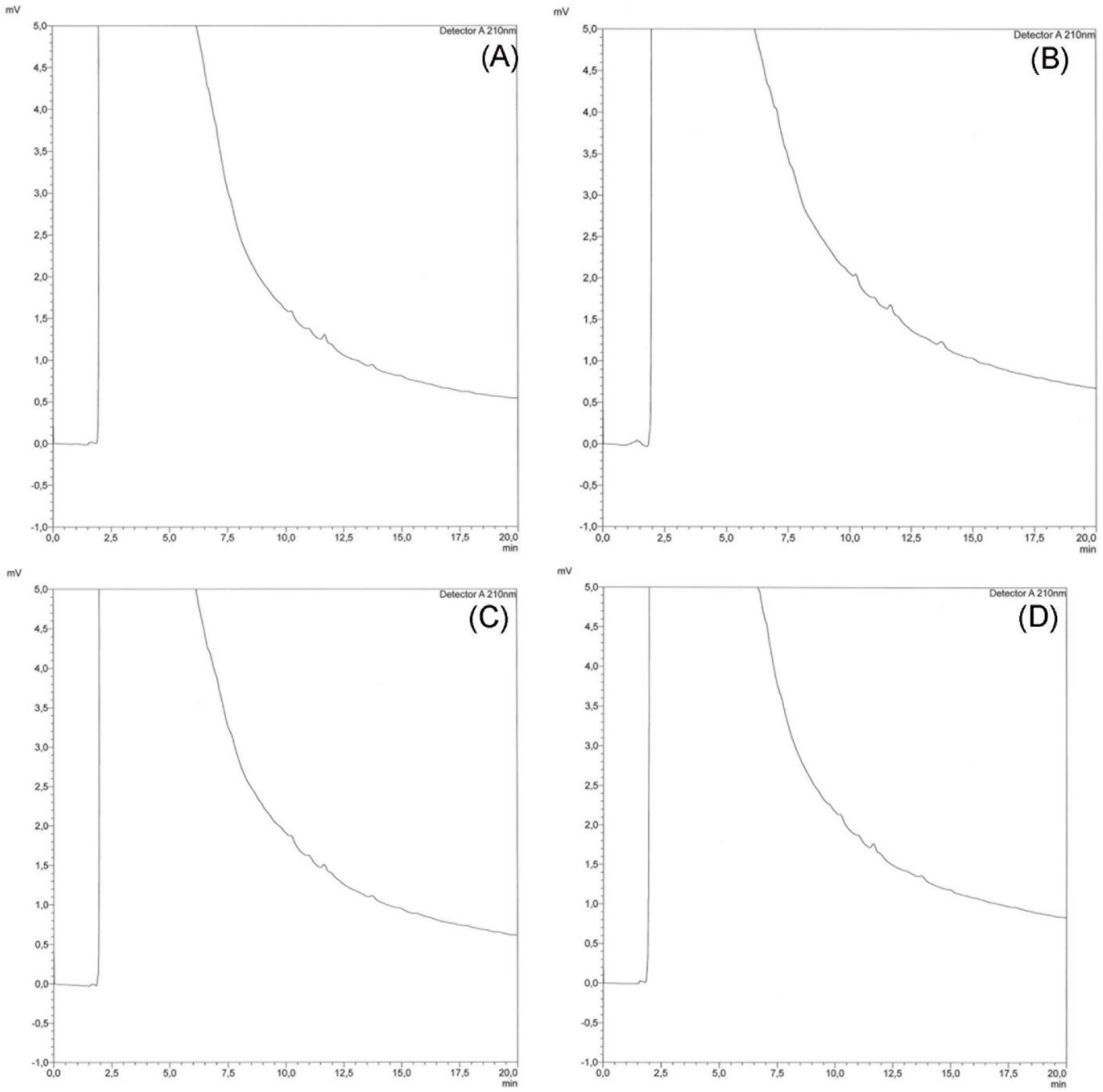

Figure S6: Chromatograms of otter no. 17 (A), of otter no. 18 (B), of otter no. 19 (C), and of otter no. 20 (D).

Chromatograms of otters under human care no. 21 - 25 used in the study

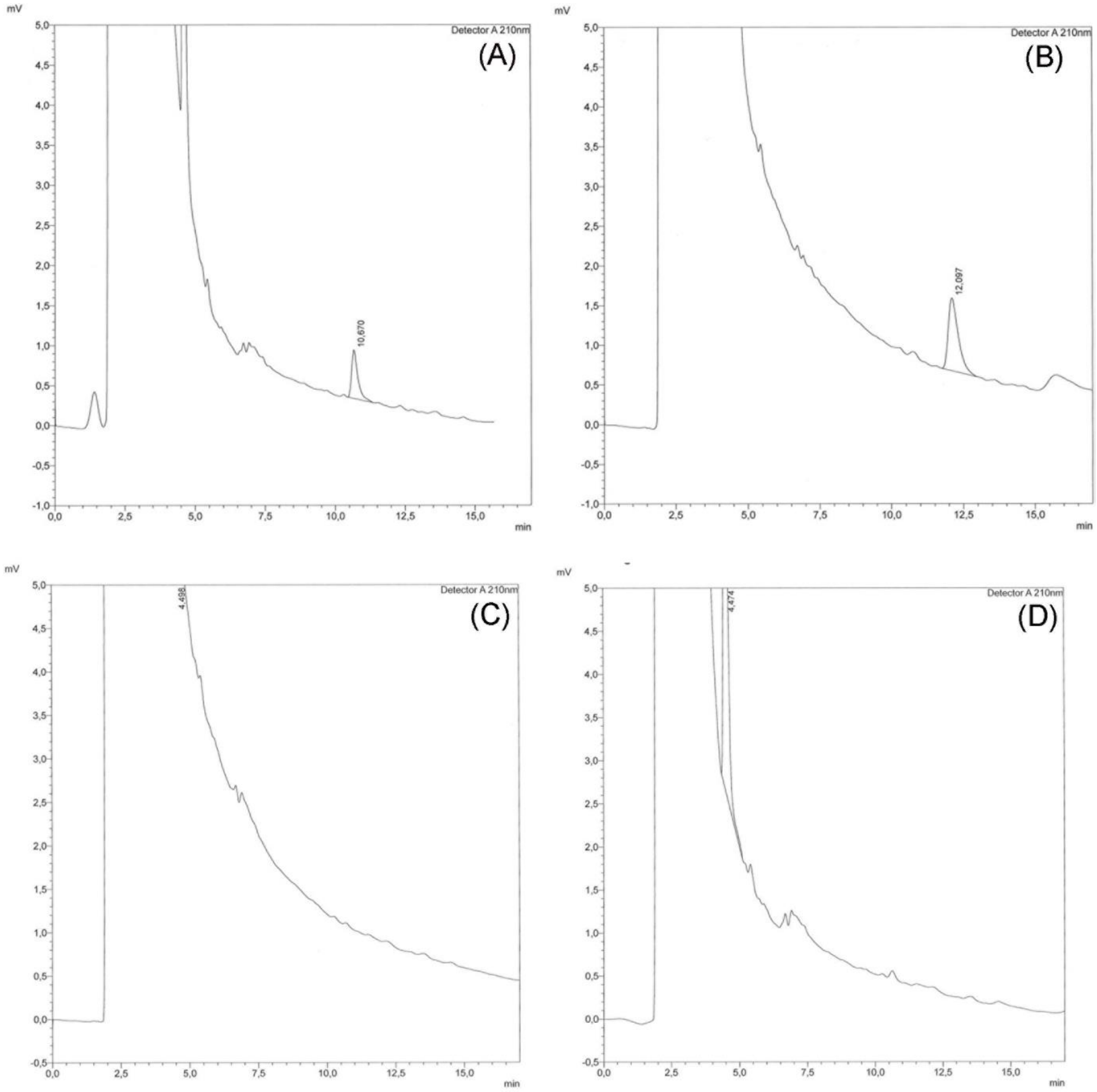

Figure S7: Chromatograms of otter no. 21 with a peak at min. 10.670 (A), of otter no. 22 with a peak at min. 12.097 (B), of otter no. 23 with a peak at min. 4.498 (C), and of otter no. 24 with a peak at min. 4.474 (D).

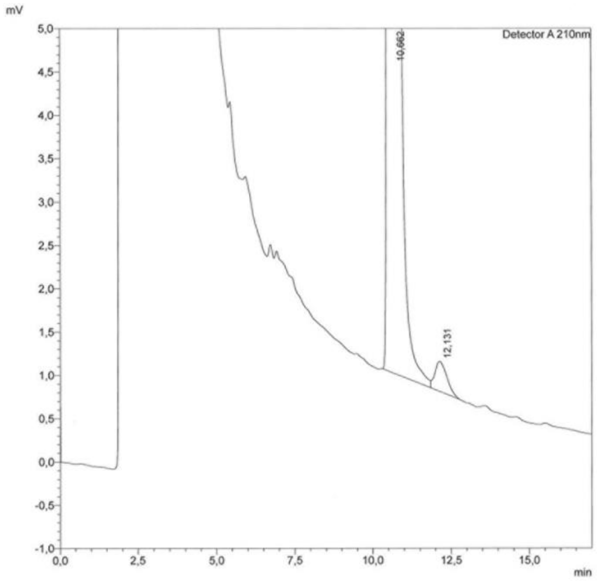

Figure S8: Chromatogram of otter no. 25 with peaks at min. 10.662 and 12.131.
